# Feasibility and Usability of Kegel Exercise Pregnancy Training App (KEPT App) among Pregnant Women with Urinary Incontinence

**DOI:** 10.3390/ijerph19063574

**Published:** 2022-03-17

**Authors:** Aida Jaffar, Noor Azimah Muhammad, Sherina Mohd Sidik, Novia Admodisastro, Rosliza Abdul Manaf, Chai Nien Foo, Nazhatussima Suhaili

**Affiliations:** 1 Department of Psychiatry, Faculty of Medicine and Health Sciences, Universiti Putra Malaysia, Serdang 43400, Malaysia; aida@upnm.edu.my; 2 Primary Care Unit, Faculty of Medicine and Defence Health, Universiti Pertahanan Nasional Malaysia, Kuala Lumpur 57000, Malaysia; 3 Department of Family Medicine, Faculty of Medicine, Universiti Kebangsaan Malaysia, Kuala Lumpur 56000, Malaysia; 4 Faculty of Computer Science & Information Technology, Universiti Putra Malaysia, Serdang 43400, Malaysia; novia@upm.edu.my; 5 Department of Community Health, Faculty of Medicine and Health Sciences, Universiti Putra Malaysia, Serdang 43400, Malaysia; rosliza_abmanaf@upm.edu.my; 6 Department of Population Medicine, Faculty of Medicine and Health Sciences, Universiti Tunku Abdul Rahman, Cheras 43000, Malaysia; foocn@utar.edu.my; 7 Klinik Kesihatan Ampang, Ministry of Health, Ampang 68000, Malaysia; drshima.suhaili@gmail.com

**Keywords:** mHealth app, pelvic floor muscle training, urinary incontinence, usability, user-centred design, pilot feasibility study

## Abstract

Pelvic floor muscle training (PFMT) is crucial to improving urinary incontinence (UI). This study aimed to assess the Kegel Exercise Pregnancy Training (KEPT) app’s feasibility and usability. This is a subgroup analysis from a researcher-blinded, randomised controlled pilot feasibility study among pregnant women with UI. The Malay version of the mHealth App Usability Questionnaire (Interactive) evaluated the app’s usability. Ten pregnant women completed the study, with mean age (SD) of 28.9 years (3.1). The app’s feasibility was rated above average. The app was reported with usable in all domains, (1) system information arrangement (4.98/7.0), (2) usefulness (4.89/7.0) and (3) ease-of-use and satisfaction (5.03/7.0). Education level was negatively correlated with the app’s feasibility (r = −0.81, *p* < 0.001) and all domains of usability such as ease-of-use (r = −0.66, *p* = 0.01), system information (r = −0.81, *p* = 0.001) and usefulness (r = −0.81, *p* = 0.001). PFMT video was among the app features chosen to be helpful. This study demonstrates that the newly developed user-centred design KEPT app is feasible and usable. However, the future app should provide direct feedback about their exercise techniques to motivate PFMT adherence.

## 1. Introduction

Urinary incontinence (UI) occurs during pregnancy when the woman experiences involuntary urine leakage [1]. The total prevalence of UI is about 41.0%, as reported by a recent meta-analysis [2] and a recent local primary care clinic study [3]. Living with this condition has been negatively affecting women’s quality of life significantly [4], implying UI is a significant public health problem even during pregnancy. Nevertheless, although pelvic floor muscle training (PFMT) can prevent and treat UI [5,6], pregnant women face several challenges to adhering to PFMT [7,8].

The mHealth app is beneficial in assisting pregnant women improved their health and health behaviour outcomes [9]. The app should be feasible (successfully used in the real world) [10] and usable (able to meet the users’ needs and requirements) [11], which is listed among the crucial categories in the mHealth app quality of standards [12]. However, not all the apps for pregnant women available in the app store are of high quality. The recent reviews demonstrated that pregnant women’s commercial apps promoting exercise were of low-to-moderate quality, with the lowest score in the behavioural impact [13,14].

Similarly, PFMT apps available in the stores were reported with credential issues, as most of them (70%, *n* = 14/20) were developed from unknown resources, and only 30% (*n* = 6/20) were developed by either doctors or physiotherapists [15]. These findings are alarming, as it is crucial to have safe apps, especially when prescribing exercise for pregnant women. Safety has recently been included as one of the vital elements in the quality standard of the mHealth app [12].

In addition to having safe and credible apps information content, the apps should influence the users. The users should be able to trust and engage with the apps; for example, women prefer using an app when a group of physicians develops a pregnancy-related health behaviour change, as it has shown its credibility. Having credible developers makes the app trustworthy, as recommended by the guideline [16]. By including the persuasive system design, the users should be able to be persuaded and engaged in using the app.

A persuasive system can be defined as “computerised software or information systems designed to reinforce, change or shape attitudes or behaviours or both without using coercion or deception” [17]. The Persuasive System Design (PSD) framework is divided into four categories: primary task support, dialogue support, system credibility support and social support [18,19]. Reviews have shown the effectiveness of some of the PSD features to improve the patient’s engagement in behavioural changes, such as supporting healthy choices in weight management [20], self-management in diabetes [21] and cardiovascular disease [22], patient empowerment [23] and caregiver support during stroke recovery [24].

However, a recent review highlighted limited evidence in the PFMT mHealth app utilising PSD for pregnant women [25]. Hence, with PSD’s involvement, this study utilises the Kegel Exercise Pregnancy Training app (KEPT app) [26] designed to empower pregnant women for PFMT adherence to improve their urinary incontinence. This study assessed the KEPT app’s feasibility and usability and its preliminary effect in improving the PFMT knowledge, attitude, practice, self-efficacy and adherence. Additionally, the severity of UI and quality of life were also analysed. This paper reports the subgroup analysis of the pilot feasibility randomised controlled trial (RCT) study [27]. This feasibility and usability study will contribute preliminary evidence of the usability properties of a KEPT app designed with some of the persuasive features that were crucial to ensure engagement from the users [28].

## 2. Materials and Methods

### 2.1. Study Design

This study was a two-arm parallel-group, researcher-blinded, pilot feasibility RCT at an urban government health clinic in Ampang, Selangor, Malaysia. This study’s preliminary effectiveness’ finding has recently been published [29]. The study participants were randomised to the intervention group that received the KEPT app and the waitlist control that received the app after completing their study. Subgroup assessments were analysed at baseline and two months after using the app. This study protocol was published recently [27] and prospectively registered with ClinicalTrials.gov (NCT04762433).

### 2.2. Participants

Pregnant women with any parity, aged 18 and above, were recruited from June 2021 to September 2021. The inclusion criteria were Malaysian citizen, Android mobile phone user with internet access and 26–27 weeks of gestation (to allow short duration between recruitment and enrolment to reduce risk of drop-out), with either stress UI or mixed UI. The potential study participants were informed with an e-poster, and those interested provided their contact number to the clinic staff to be contacted by the research team. The pilot RCT obtained ethics approval from the Ethics Committee for Research Involving Human Subjects, Universiti Putra Malaysia (JKEUPM-2019-368) Medical Research and Ethics Committee (MREC), Ministry of Health Malaysia (NMRR-19-412-45606) in August 2019. All study participants invited to this study complied with the Declaration of Helsinki [30]. Before the study commencement, they were required to sign an online consent form.

### 2.3. Intervention: KEPT App (Interactive Android)

**Figure 1:**
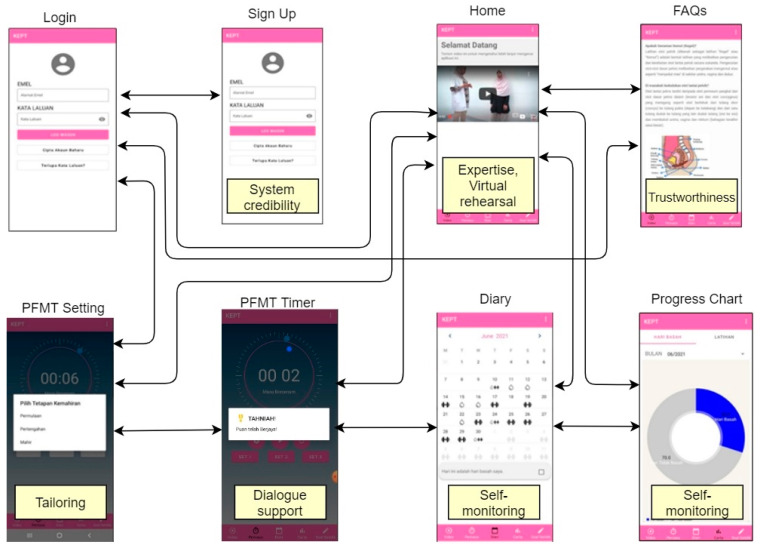
The KEPT app wireflow diagram with its persuasive design.

**Figure 2:**
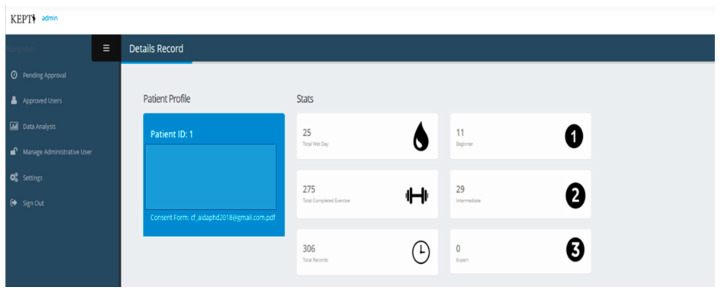
User detail PFMT app record (KEPT web).

Participants were allocated to the intervention group (while continuing antenatal follow-up as usual) with an eight-week KEPT app consisting of a PFMT educational video, training timer, progress chart, daily reminder notification, and frequently asked questions (Figure 1). This app was developed from a user-centred design, adopting the PFMT techniques from an evidence-based PFMT programme using educational video [31]; it has been validated [26] and has undergone expert usability testing [32]. It has been upgraded from the standalone version to an interactive version whereby the researchers could monitor their activities and their UI symptoms progression (Figure 2).

### 2.4. Outcome Measures

This article was intended to report the sub-analysis of the intervention group. The primary outcome was the feasibility of the KEPT app to determine the satisfaction of pregnant women using the app [27,33]. The KEPT app feasibility was defined as the extent of the potential success of the app in a primary care clinic [10]; the questionnaires used are listed in Table 1.

The assessment of the usability element includes ease of use and satisfaction (8 items), system information arrangement (6 items) and usefulness (7 items) [34] from the validated questionnaire used for the usability was the Malay version of the mHealth Application Usability Questionnaire (MAUQ) interactive version. The original MAUQ (interactive version) has undergone the backwards-forward translation process and cognitive debriefing to ensure the validity of the questionnaire. The reliability analysis of the Malay version was excellent, with a Cronbach alpha of 0.991.

The preliminary effect on the severity of urinary incontinence, quality of life, PFMT knowledge, attitude, practice, self-efficacy and adherence was assessed (Table 1). Subsequently, the study participants were asked to choose the best three features of the app, which assisted them in performing the PFMT regularly.

**Table 1:** Study outcomes.

Outcome	Description
Feasibility KEPT app	To assess the feasibility of the app after using the app for two months. (1) How many stars would you use to recommend this app to your friends? (2) How many stars would you rate the content of this app? (3) How many stars would you rate the comfort of using this app? (4) How many stars would you rate simplicity using this app? (5) How many stars would you rate the recruitment process using this app? (6) How many stars would you rate the quality of the app? (7) Factors influenced/hindered using this app? (8) What motivates you to continue the PFMT?
Usability KEPT app	To assess the app’s usability using the Malay version of the mHealth Application Usability Questionnaire (MAUQ) interactive after using the apps for two months.
Urinary incontinence	To assess the severity of urinary incontinence symptoms at baseline and two months post interventions using the International Consultation on Incontinence Questionnaire-Urinary Incontinence Short Form (ICIQ-UI SF) [35,36].
Quality of life	To assess the quality of life among pregnant women with UI at baseline, and two months post interventions. International Consultation on Incontinence Questionnaire Urinary Incontinence-Lower Urinary Tract Symptom quality of life (ICIQ-LUTSqol) [35,36].
PFMT knowledge, attitude and practice	To assess the knowledge, attitude and practices towards PFMT at baseline, two months post interventions, using the Knowledge, Attitude and Practice towards Pelvic Floor Muscle Training [37]
PFMT self-efficacy	To measure the self-efficacy score at baseline, two-months post-intervention using the Self-Efficacy Scale For Practicing Pelvic Floor Exercise Questionnaire (SESPPFE) [38]
PFMT adherence	To assess the PFMT adherence at baseline and two months post interventions using the Exercise Adherence Rating Scale (EARS) [39].

PFMT—pelvic floor muscle training.

### 2.5. Sample Size

This study was expected to have 64 participants within two months as there was no requirement to have a powered sample size for the pilot study [40]. A minimum of twelve and up to 30 participants for each group was considered appropriate in feasibility studies [41] and pilot studies [42].

### 2.6. Randomisation and Blinding

The randomisation process was conducted with stratification at their parity categories using the randomisation app [43]. Only researchers were blinded in the group allocation, as study participants were challenging to be blinded to the intervention. Allocation to the group was using the sealed envelope by the clinic staff.

### 2.7. Statistical Methods

All analyses were performed utilising the Statistical Package for the Social Sciences version 27.0 [44,45]. Data are presented according to normality testing (Shapiro–Wilk test) distribution, with the kurtosis and skewness [46] with mean and standard deviation (SD) or median, interquartile range (IQR) for continuous variables and counts (percentages) for categorical variables. Pearson and Spearman Rank correlation analyses were conducted to determine the relationship between two variables. Baseline characteristics of the participants and the study outcomes were determined using the paired T-test. The one-group preliminary effect was conducted as per-protocol analysis without imputation. All analyses with a *p*-value <0.05 were considered statistically significant.

## 3. Results

### 3.1. Participant Characteristics

Ten participants from Malay ethnicity from low socioeconomic status have completed this study. Their mean age (SD) was 28.9 years (3.1), median Body Mass Index (BMI) was 29.5 kgm^2^ (Interquartile range of 7.8) and 70% (*n* = 7/10) of them were multigravida and had Stress UI. The correlation between sociodemographic with feasibility and usability is listed in Table 2. This finding suggests a strong negative correlation between the level of education and the app’s feasibility with the Spearman correlation coefficient (*r* = −0.81, *p* < 0.001). Similarly, levels of education were negatively correlated with ease-of-use (*r* = −0.66, *p* = 0.01), system information (*r* = −0.81, *p* = 0.001) and usefulness (*r* = −0.81, *p* = 0.001). Hence, pregnant women at any level of education can use this app.

### 3.2. Feasibility of the KEPT App

Pregnant women rated 3–4 stars of the app’s feasibility (Table 3). They described several difficulties or barriers to using the app, such as time limitation (*n* = 3), full storage use (*n* = 1) and personal issues (*n* = 1). Study participants listed their motivation for using the app, such as a benefit for their baby (*n* = 1), improving their UI symptoms (*n* = 3), improving vaginal muscle (*n* = 1), healthy (*n* = 1) and easing the process of her childbirth (*n* = 1).

### 3.3. Usability of the KEPT App

The study participants neither agree nor disagree with KEPT app’s system information arrangement (4.98/7.0) and usefulness (4.89/7.0). They rated with somewhat agree with the app’s ease-of-use and satisfaction (5.03/7.0). The breakdown of each item is listed in Table 4.

### 3.4. The Preliminary Effect of the KEPT App

After using the app, the study outcomes (Table 5) among the study participants showed a significant improvement in their UI symptoms with a mean difference of 1.9 (95% CI 0.23–3.57).

### 3.5. KEPT App Features That Assisted Them to Perform PFMT

Study participants were asked to choose the best three app features that help them adhere to the PFMT. All of them (100%, *n* = 10/10) chose the PFMT video as the first in their list, half of them selected the pop-up notification reminder (50%, *n* = 5/10) and only two (20%, *n* = 2/10) stated that the system credibility and frequently asked questions had helped them (Table 6).

## 4. Discussion

This study reported that the KEPT app, which builds in an interactive version, was feasible, simple-to-use and useful. A significant improvement in the severity of UI symptoms was demonstrated. Subsequently, the video, reminder, and self-monitoring were chosen as the “persuasive” elements, which assisted them in performing PFMT as their routine. Study participants improved significantly in their PFMT practices and quality of life.

Pregnant women were influenced using the app, as they appreciated its usefulness and had satisfaction using it. Everyone appreciated the video delivered by the app, as it was demonstrated by the physiotherapist and has undergone validation study (face validity among the experts) and produced by the tertiary hospital. Similarly, an online educational video developed by experts in antenatal colostrum expression reported increased knowledge and confidence among their study participants [47]. This study supported that using an educational PFMT video was acceptable to educate and improve their confidence in performing their daily pelvic exercise. Additionally, this study may suggest that any education level can use this app effectively, despite the previous study highlighting that those with a lower level of education were less likely to engage with mHealth interventions than those with a higher education level [48].

The training timer was not favourable when compared to the remainder. This was another interesting finding to the researchers, as the team had spent most of their focus in designing and re-designing the training timer to ensure better engagement from the pregnant women. The finding was probably due to the time needed for them to continue using the timer as the minimum duration was 80 s and the maximum duration was almost 160 s. Being pregnant, women have reported busy doing their house chores or working activities, which hindered them from performing the exercise [7]. The time factor could be one of the reasons for the unfavoured training timer in this study.

A significant improvement in the UI symptoms after two months may improve their quality of life if using it for a longer duration. Using the app offers the opportunity and flexibility in empowering pregnant women to adopt PFMT into their busy daily schedules. This finding was supported by another audio-based app study that involved pregnant women (nulliparous) and demonstrated improved severity of the UI during their post-partum [49]. This pilot study added new evidence that the PFMT app can be used for self-empowerment among low-income pregnant women with limited access to other health resources.

Finally, this study provides an eye-opener to the researcher’s team. As harnessing the user-centred approach in developing the app, this study reported that it should consider improvising the training timer interface to attract the user and reduce their time. Other strategies are conducting qualitative research among the lower socioeconomic pregnant women and understanding their views without compromising the objectives of the apps.

### Limitation of the Study

This study involved only pregnant women from the lower socioeconomic status, which did not represent the Malaysia study population. Despite the majority receiving their tertiary education being in the lower group in the economic status, other issues need to be explored, for example, financial issues, stress issues and time issues to manage the family. Subsequently, the app’s persuasiveness and feasibility should be assessed using a validated questionnaire that can measure the app’s level of persuasion and feasible properties. It is recommended to conduct a qualitative study to follow up with the study participants and understand their facilitators or modifier factors. This study sample size of 10 has a post hoc power of 0.8 and is able to detect 0.9 of effect size between the null hypothesis and the alternative hypothesis with a one-tail significance level of 0.05, using the G-Power calculation ver 3.9.1.4. Hence, the sample size included in the analysis was adequate.

## 5. Conclusions

The KEPT app, developed from a user-centred design and behavioural change theory and accompanied by the persuasive system design, was feasible and usable for pregnant women of lower socioeconomic status. Educational video with the virtual rehearsal persuasive design was the most preferred in assisting them to engage with the app. This app enables pregnant women to empower themselves in self-managing their UI. The performance feedback is an essential persuasive strategy to be embedded in the future app to improve the retention rate. However, as women need to use it daily, it is challenging to keep their interest for long-term use. Future study is warranted to refine the app’s prototype to ensure the users’ engagement from all socioeconomic status groups available in Malaysia.

## Figures and Tables

**Table 2:** Correlation of demographic characteristics with feasibility and usability.

	Age	Level of Education	Ease of Use	System Information	Usefulness	Feasibility
Age	1	−0.613	0.299	0.427	0.379	0.394
Level of education ^#^	−0.613	1	−0.656 *	−0.805 **	−0.805 **	−0.813 **
Ease of use	0.299	−0.656 *	1	0.949 **	0.839 **	0.545
System information	0.427	−0.805 **	0.949 **	1	0.959 **	0.501
Usefulness	0.379	−0.805 **	0.839 **	0.959 **	1	0.456
Feasibility	0.394	−0.813 **	0.545	0.501	0.456	1

* Correlation is significant at the 0.05 level (2-tailed). ** Correlation is significant at the 0.01 level (2-tailed). ^#^ Spearman Rank.

**Table 3:** Feasibility KEPT app.

Statements	Median (IQR)
(1) How many stars would you rate to recommend this app to your friends?	4.0 (2)
(2) How many stars would you rate the quality content of this app?	3.7 (1.25) *
(3) How many stars would you rate the comfort of using this app?	4.0 (2)
(4) How many stars would you rate the simplicity of using this app?	4.0 (2)
(5) How many stars would you rate using this app’s recruitment process?	3.8 (2)
(6) How many stars would you rate the app’s quality?	3.9 (2)

* Mean (SD) (Shapiro–Wilk test for normality testing).

**Table 4:** The usability of the KEPT app.

Statements	Score Mean (SD)
The app was easy to use.	4.9 (0.99)
It was easy for me to learn to use the app.	5.1 (0.88)
I like the interface of the app.	5.0 (1.1)
The information in the app was well organised, so I could easily find the information I needed.	5.1 (0.99)
I feel comfortable using this app in social settings.	5.1 (0.99)
The amount of time involved in using this app has been fitting for me.	4.9 (1.2)
I would use this app again.	5.0 (1.2)
Overall, I am satisfied with this app.	5.1 (0.99)
Whenever I made a mistake using the app, I could recover easily and quickly.	5 (1.1)
This mHealth app provides an acceptable way to receive healthcare services.	5 (1.3)
The app adequately acknowledged and provided information to let me know the progress of my action.	4.9 (1.2)
The navigation was consistent when moving between screens.	4.9 (1.2)
The interface of the app allowed me to use all the functions (such as entering information, responding to reminders, viewing information) offered by the app.	5.1 (0.99)
This app has all the functions and capabilities I expected it to have.	5 (1.1)
The app would be useful for my health and well-being.	5.1 (0.99)
The app improved my access to healthcare services.	4.8 (1.4)
The app helped me manage my health effectively.	4.9 (1.2)
The app made it convenient for me to communicate with my healthcare provider.	4.8 (1.4)
Using the app, I had many more opportunities to interact with my healthcare provider.	4.8 (1.4)
I felt confident that any information I sent to my provider using the app would be received.	4.8 (1.4)
I felt comfortable communicating with my healthcare provider using the app.	5.0 (1.1)

1—strongly disagree; 2—disagree; 3—somewhat disagree; 4—neither agree nor disagree; 5—somewhat agree; 6—agree; 7—strongly agree.

**Table 5:** Effect of the KEPT app on the study outcomes.

Variable	Baseline Mean (SD)	Post-Intervention Mean (SD)	95% CI	*p*-Value
PFMT knowledge	9.10 (3.51)	11.00 (2.31)	−4.46–0.66	0.127
PFMT attitude	27.40 (8.54)	32.90 (4.58)	−13.61–2.61	0.159
PFMT practice	8.90 (3.38)	10.30 (2.66)	−2.99–0.19	0.077
PFMT self-efficacy	42.35 (25.55)	60.58 (18.14)	11.33–43.86	0.142
PFMT adherence	14.30 (5.47)	12.00 (4.29)	−3.01–7.61	0.353
Severity UI	8.10 (2.31)	6.20 (2.78)	0.23–3.57	0.03
Quality of life	30.80 (7.33)	27.80 (7.58)	−0.55–6.50	0.085

CI—confidence interval; PFMT—pelvic floor muscle training.

**Table 6:** The features of the KEPT app and the Persuasive System Design.

KEPT App	Persuasive System Design	Users (*N* = 10)
Educational video by a registered physiotherapist with a “model”	System credibility Expertise and authority	10 (100)
Training timer according to the user’s confidence and capability	Primary support Tailoring	4 (40.0)
The KEPT app produced by the university	System credibility Trustworthiness	2 (20.0)
Frequent asked questions (FAQs) to provide further information	Primary support Tailoring	2 (20.0)
Calendar charting of the UI symptoms	Primary task Self-monitoring	3 (30.0)
Regular daily reminder to perform PFMT as their routine behaviour	Dialogue support Reminder	5 (50)

## Data Availability

The data presented in this study are available on request from the corresponding author. The data are not publicly available due to privacy.
